# The influence of marital status on survival of gallbladder cancer patients: a population-based study

**DOI:** 10.1038/s41598-017-05545-0

**Published:** 2017-07-13

**Authors:** Xinxing Li, Ye Liu, Yi Wang, Canping Ruan, Haolu Wang, Xiaowen Liang, Yanping Sun, Zhiqian Hu

**Affiliations:** 1 Department of General Surgery, Changzheng Hospital, the Second Military Medical University, 415S, Fengyang Road, Shanghai, 200003 China; 2 Department of Blood Transfusion, Changzheng Hospital, the Second Military Medical University, 415S, Fengyang Road, Shanghai, 200003 China; 3 Therapeutics Research Centre, Faculty of Medicine, The University of Queensland, Woolloongabba, QLD 4102 Australia

## Abstract

Marital status has been found to be a prognostic factor for survival in various cancers, but its role in gallbladder cancer (GBC) has not been fully studied. In this study, we used the Surveillance, Epidemiology, and End Results Program (SEER)-registered database to analyze the survival of GBC patients with different marital status. A total of 6,627 GBC patients were selected from SEER database from 2004 to 2013. The age, race, grade, histologic type, AJCC stage, SEER stage and marital status were identified as independent prognostic factors. Married GBC patients had a higher 5-year cancer-specific survival (CSS) than that of unmarried ones (20.1% v.s. 17.8%, P < 0.05). Subgroup analyses showed that widowed patients had 14.0% less of 5-year CSS compared to married ones of stage I (55.9% v.s. 41.9%, P < 0.05), 14.7% of stage II (15.6% v.s. 10.9%, P < 0.05), and 1.5% of stage III + IV (2.9% v.s. 1.4%, P < 0.05). In addition, single is an independent prognostic factor at stage III + IV (HR = 1.225, 95%CI 1.054–1.423, P = 0.008). These results indicated that widowed patients were at a high risk of cancer-specific mortality and marriage can be a protective prognostic factor in CSS.

## Introduction

Gallbladder cancer (GBC) is one of the most common malignant cancer in biliary system^1^. Despite many advances in the diagnosis and treatment of this disease, the prognosis of GBC is still poor, with less than 5% of 5-year survival^1–3^. Studies have shown that the marital status has significant impacts on the survival of various cancers, including colorectal^4^, gastric^5, 6^, pancreatic^7^ and tracheal^8^ cancer. Married individuals have better prognosis and lower mortality of major causes of death compared to those never married, separated, widowed, or divorced^9–11^. Furthermore, marital status has been demonstrated as an independent prognostic factor of survival in various cancers^12–15^. Li *et al*. reported that unmarried patients were at greater risk of cancer specific mortality while widowed patients were at the highest risk of death compared to other groups, which were analyzed in 112, 776 colorectal cancer patients selected from SEER data^4^. Jin *et al*. demonstrated that marriage had a protective effect against undertreatment and cause-specific mortality in gastric cancer^5^. But few study focused on the influence of marital status on the survival of GBC patients. Therefore, we systematically investigated the effect of marital status on the clinicopathological features and survival of GBC patients in this study.

## Results

### Patient characteristics

A total of 6,627 GBC patients between 2004 and 2013 were selected from SEER database, including 1,959 males and 4,668 females. Among the 6,627 patients, the average age is 70 (range of 21–104). 3451 patients are married; 686 are divorced or separated; 1586 are widowed and 904 are single. The demographic and characteristics of patients were summarized in Table 1. The marital status of patients is correlated to sex, age, race, histologic type, AJCC stage and SEER stage in GBC (p < 0.05).

**Table 1:** Characteristics of patients with GBC in SEER database.

Parameter	Characteristic	N	Married	Divorced/ Separated	Widowed	Single	χ2	P value
			N(%)	N(%)	N(%)	N(%)		
Sex								
	Male	1959	1356(39.29)	160(23.32)	172(10.84)	271(29.98)	436.672	P < 0.001
	Female	4668	2095(60.71)	526(76.68)	1414(89.16)	633(70.02)		
Age								
	<60	1651	999(28.95)	225(32.80)	62(3.91)	365(40.38)	542.421	P < 0.001
	≥60	4976	2452(71.05)	461(67.20)	1524(96.09)	539(59.62)		
Race								
	White	5055	2661(77.11)	517(75.36)	1238(78.06)	639(70.69)	164.611	P < 0.001
	Black	802	311(9.01)	119(17.35)	177(11.16)	195(21.57)		
	*Others	748	468(13.56)	49(7.14)	168(10.59)	63(6.97)		
	Unknow	22	11(0.32)	1(0.15)	3(0.19)	7(0.77)		
Grade								
	Well differentiated	685	368(10.66)	60(8.75)	167(10.53)	90(9.96)	19.991	P = 0.067
	Moderately differentiated	1982	1028(29.79)	198(28.86)	475(29.95)	281(31.08)		
	Poorly differentiated	1853	995(28.83)	190(27.70)	409(25.79)	259(28.65)		
	Undifferentiated	122	76(2.20)	12(1.75)	22(1.39)	12(1.33)		
	Unkown	1985	984(28.52)	226(32.94)	513(32.34)	262(28.98)		
Histologic type								
	Adenomas adenocarcinomas	5388	2850(82.58)	552(80.47)	1263(79.63)	723(79.98)	63.583	P < 0.001
	Epithelial neoplasms	435	217(6.29)	42(6.12)	115(7.25)	61(6.75)		
	Cystic, mucinous and serous neoplasms	371	189(5.48)	41(5.98)	82(5.17)	59(6.52)		
	Unspecified neoplasms	151	46(1.33)	27(3.93)	67(4.23)	11(1.22)		
	#Others	282	149(4.32)	24(3.50)	59(3.72)	50(5.53)		
AJCC Stage								
	I	1755	890(25.79)	165(24.05)	465(29.32)	235(26.00)	50.702	P < 0.001
	II	1872	984(28.51)	185(26.97)	445(28.06)	258(28.54)		
	III	173	93(2.70)	23(3.35)	34(2.14)	23(2.54)		
	IV	2351	1283(37.18)	265(38.63)	486(30.64)	317(35.07)		
	UNK Stage	476	201(5.82)	48(7.00)	156(9.84)	71(7.85)		
SEER Stage								
	Localized	2133	1049(30.40)	199(29.01)	594(37.45)	291(32.19)	67.165	P < 0.001
	Regional	1584	857(24.83)	161(23.47)	341(21.50)	225(24.89)		
	Distant	2675	1456(42.19)	296(43.15)	563(35.50)	360(39.82)		
	UNK Stage	235	89(2.58)	30(4.37)	88(5.55)	28(3.10)		

*Other includes American Indian/AK Native, Asian/Pacific Islander.

^#^Other includes squamous cell carcinoma/complex epithelial neoplasms/complex mixed and stromal neoplasms.

/ductal and lobular neoplasms.

Compared to married, divorced/separated and single patients with GBC, widowed group had higher proportion of women (89.16% VS 60.71%, 76.68% and 70.02%), more prevalence of elderly patients (96.09% VS 71.05%, 67.20% and 59.62%), higher percentage of AJCC stage I/II (57.38% VS 54.30%, 51.02% and 54.54%) and of SEER localized stage (37.45% VS 30.40%, 29.01% and 32.19%) All these differences were statistically significant (Table 1, P < 0.05).

### Effect of marital status on CSS in the SEER database

**Figure 1:**
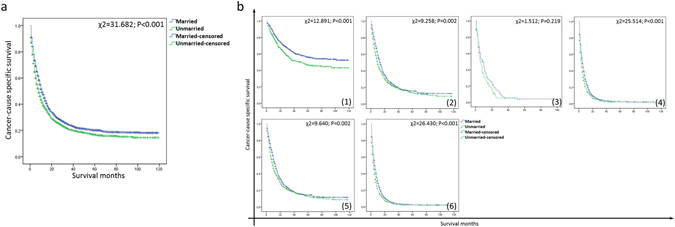
Survival curves of married and unmarried GBC patients. (**a**) All stages: χ2 = 31.682, P < 0.001; (**b**) (1) stage I: χ2 = 12.891, P < 0.001; (2) stage II: χ2 = 9.258, P = 0.002; (3) stage III: χ2 = 1.512, P = 0.219; (4) stage IV: χ2 = 25.514, P < 0.001; (5) stage II + III: χ2 = 9.640, P = 0.002; (6) stage III + IV: χ2 = 26.430, P < 0.001.

**Figure 2:**
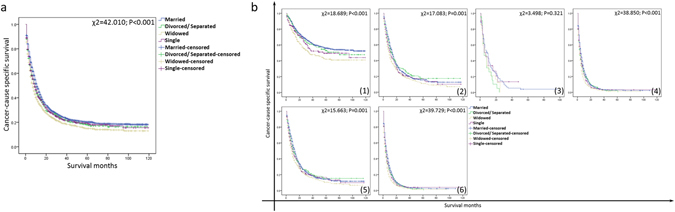
Survival curves of GBC patients with different marital status (married, divorced/separated, widowed and single). (**a**) All stages: χ2 = 42.010, P < 0.001; (**b**) (1) stage I: χ2 = 18.698, P < 0.001; (2) stage II: χ2 = 17.083, P = 0.001; (3) stage III: χ2 = 3.498, P = 0.321; (4) stage IV: χ2 = 38.850, P < 0.001; (5) stage II + III: χ2 = 15.663, P = 0.001; (6) stage III + IV: χ2 = 39.729, P < 0.001.

Married patients showed a higher 5-year CSS compared to unmarried patients (Fig. 1, 20.1% v.s. 17.8%, P < 0.001). Married group had higher survival rate in patients of TNM stage I, II and IV (stage I: χ2 = 12.891, P < 0.001; stage II: χ2 = 9.258, P = 0.002; stage IV: χ2 = 25.514, P < 0.001). This difference was not significant for stage III because of the small number of patients (stage III: χ2 = 1.512, P = 0.219). There were significant differences between married group and unmarried group of stage II + III patients (χ2 = 9.640, P = 0.002) and stage III + IV patients (χ2 = 26.430, P < 0.001). A shown in Fig. 2, the subgroup analysis of marital status (married, widowed, divorced/separated and single) confirmed these findings.

Married group had the highest 3-year and 5-year CSS (24.3% and 20.1%) compared to divorced/separated group (22.4% and 18.1%), single group (22.3% and 19.2%), and widowed group (19.1% and 14.9%, P < 0.05). While the CSS of the married group was higher than the single group (P < 0.05). Additionally, age, race, grade, histologic type, AJCC stage, SEER stage and marital status were identified as significant risk factors for the survival of GBC on univariate analysis (Table 2, P < 0.05). All these seven variables were independent prognostic factors in multivariate analysis of Cox regression (Table 2, P < 0.05).

**Table 2:** Univariate and multivariate survival analysis for evaluating the influence of marital status on CSS of GBC patients in SEER database.

Parameter	Characteristic	3-year CCS	5-year CCS	Univariate analysis		Multivariate analysis	
				Log rank χ2 test	*P*	HR(95%CI)	*P*
Sex				3.011	0.083		NI
	Male	20.6%	16.5%				
	Female	23.5%	19.4%				
Age				41.777	0.000		0.000
	<60	27.0%	23.2%			Reference	
	≥60	21.1%	17.0%			0.708(0.658–0.762)	
Race				19.311	0.000		0.005
	White	23%	18.7%			Reference	
	Black	17.2%	14.7%			3.259(1.234–8.795)	
	*Others	25%	21.3%			3.709(1.386–9.930)	
	Unkown	30.4%	22.4%			3.226(1.204–8.641)	
Grade				753.005	0.000		0.000
	Well differentiated	52.4%	44.9%			Reference	
	Moderately differentiated	32%	25.9%			0.552(0.481–0.633)	
	Poorly differentiated	15%	11.7%			0.672(0.617–0.732)	
	Undifferentiated	20.3%	16.8%			0.991(0.915–1.073)	
	Unkown	11.4%	9.7%			0.889(0.656–1.204)	
Histologic type				131.039	0.000		0.004
	Adenomas; adenocarcinomas	24.3%	19.8%			Reference	
	Epithelial neoplasms	16.3%	15.1%			0.768(0.666–0.885)	
	Cystic, mucinous and serous neoplasms	17.7%	14.3%			0.771(0.644–0.923)	
	Unspecified neoplasms	10.4%	7.5%			0.824(0.686–0.989)	
	#Others	14.6%	12.6%			0.873(0.680–1.122)	
AJCC Stage				1891.004	0.000		0.000
	I	58.7%	51.6%			Reference	
	II	20.6%	14.8%			0.384(0.318–0.464)	
	III	5.1%	3.9%			0.814(0.684–0.769)	
	IV	3.4%	2.5%			1.029(0.800–1.323)	
	UNK Stage	15%	11.2%			1.288(1.041–1.594)	
SEER				1807.983	0.000		0.000
	Localized	52.7%	45.4%			Reference	
	Regional	20.3%	14.9%			0.688(0.544–0.870)	
	Distant	3.7%	2.9%			0.895(0.708–1.132)	
	UNK Stage	10%	4.6%			1.198(0.922–1.557)	
Marital Status				42.010	0.000		0.000
	Married	24.3%	20.1%			Reference	
	Divorced/ Separated	22.4%	18.1%			0.864(0.787–0.948)	
	Widowed	19.1%	14.9%			0.942(0.832–1.065)	
	Single	22.3%	19.2%			1.087(0.928–1.208)	

*Other includes American Indian/AK Native, Asian/Pacific Islander. ^#^Other includes squamous cell carcinoma/complex epithelial neoplasms/complex mixed and stromal neoplasms/ductal and lobular neoplasms. NI: not included in the multivariate survival analysis.

### Effect of marital status on CSS stratified by gender, age and race

**Figure 3:**
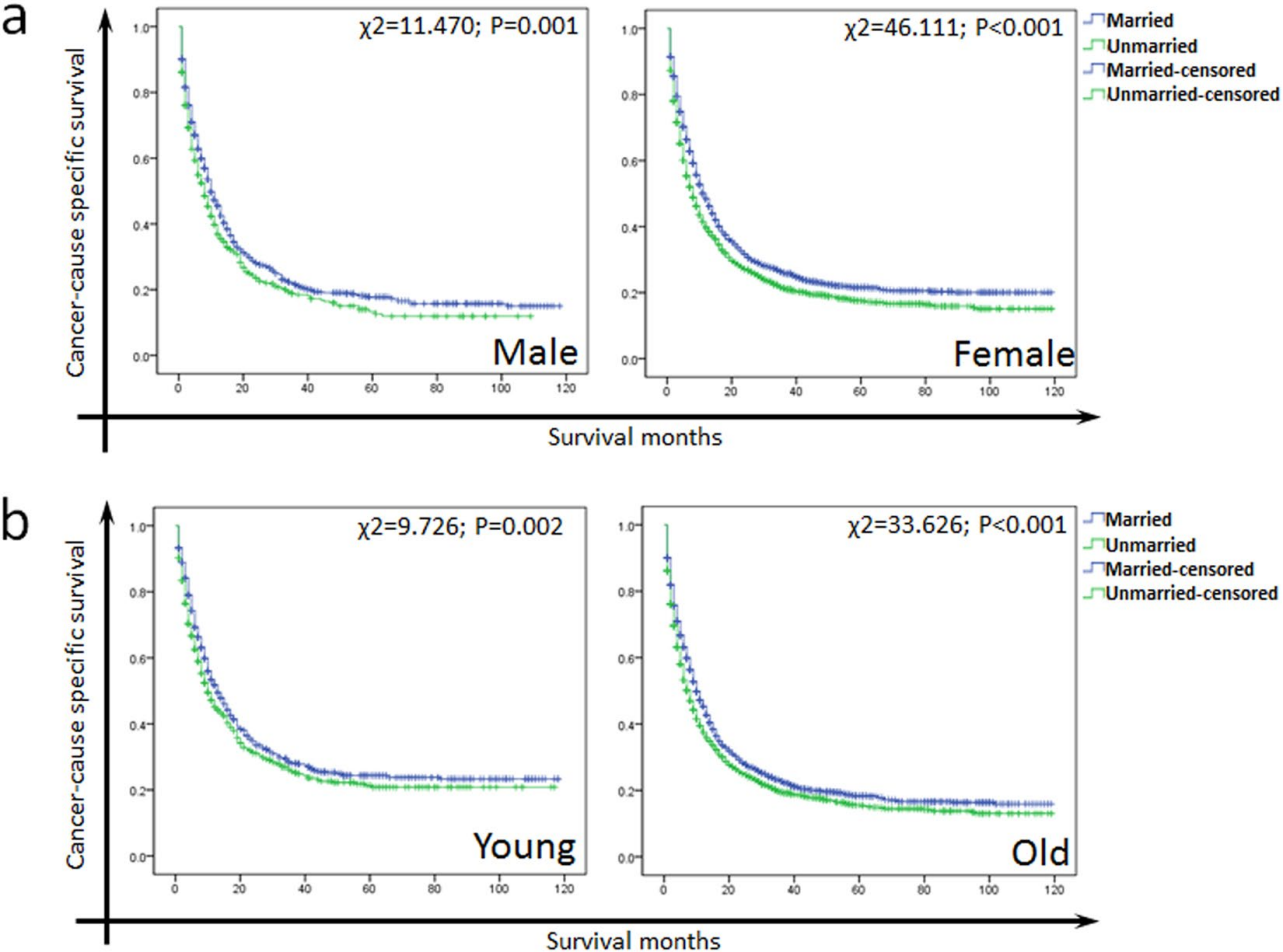
(**a**) The survival curves of male and female GBC patients with different marital status. (**b**) The survival survival curves of young and old GBC patients with different marital status.

**Figure 4:**
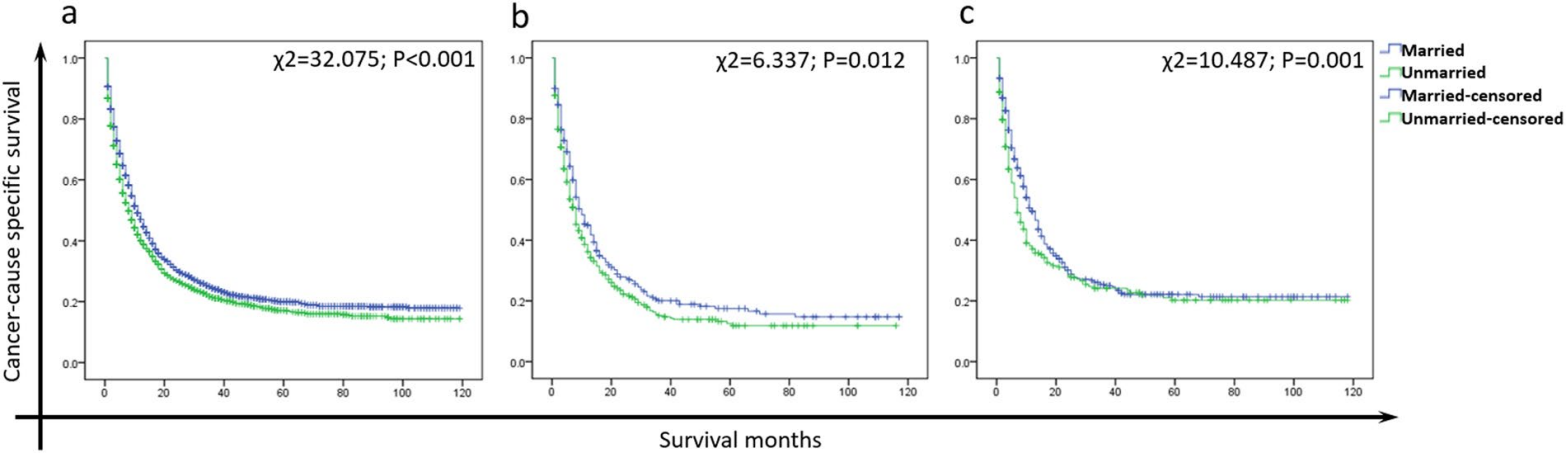
The survival curves of white (**a**), black (**b**) and other (**c**) GBC patients with different marital status. (**c**) American Indian/AK Native, Asian/Pacific Islander.

We further explored the effect of marital status on CSS stratified by gender, age and race. As shown in Fig. 3a, married ones had a better survival compared to unmarried in both males (χ2 = 11.470, P = 0.001) and females (χ2 = 46.111, P < 0.001). Compare to unmarried patients, married patients with different ages and races all had better survival (Fig. 3b and Fig. 4, P < 0.05).

### Subgroup analysis for evaluating the effect of marital status on each TNM stage

According to the univariate analysis, marital status was related to the survival of AJCC stage I (Table 3, χ^2^ = 18.689, P < 0.001), stage II (χ2 = 17.083, P = 0.001) and stage III + IV subgroups (χ2 = 39.729, P < 0.001). We also found that widowed patients had 14.0% less of 5-year CSS compared to married patients of stage I (55.9% VS 41.9%, P < 0.05), 14.7% of stage II (15.6% VS 10.9%, P < 0.05), and 1.5% of stage III + IV (2.9% VS 1.4%, P < 0.05). Single was an independent prognostic factor for stage III + IV patients (Table 3, HR = 1.225, 95%CI 1.054–1.423, P = 0.008).

**Table 3:** Univariate and multivariate analysis of marital status on CSS of GBC patients based on TNM stage.

**Parameter**	**Characteristic**	**3-year CCS**	**5-year CCS**	**Univariate analysis**		**Multivariate analysis**	
				**Log rank χ2 test**	**P value**	**HR(95%CI)**	**P value**
**TNM Stage**							
**Stage I**							
Marital Status				18.689	0.000		
	Married	63.5%	55.9%			Reference	
	Divorced/Separated	61.0%	52.8%			0.802(0.622–1.034)	0.089
	Widowed	48.3%	41.9%			0.890(0.626–1.263)	0.513
	Single	54.3%	49.5%			1.224(0.930–1.611)	0.148
**Stage II**							
Marital Status				17.083	0.001		
	Married	21.3%	15.6%			Reference	
	Divorced/ Separated	23.9%	17.4%			0.870(0.735–1.030)	0.105
	Widowed	17.6%	10.9%			0.860(0.681–1.087)	0.208
	Single	20.6%	17.0%			1.132(0.938–1.365)	0.196
**Stage III **+ **IV**							
Marital Status				39.729	0.000		
	Married	3.7%	2.9%			Reference	
	Divorced/ Separated	3.3%	2%			0.886(0.777–1.010)	0.069
	Widowed	2.3%	1.4%			0.996(0.840–1.182)	0.965
	Single	3.9%	3.9%			1.225(1.054–1.423)	0.008

P-values refer to comparisons between two groups and were adjusted for age, race, grade and histologic type as covariates.

NI: not included in the multivariate survival analysis.

## Discussion

Marital status has been found to be a prognostic factor for survival in various cancers. Mahdi *et al*. selected 49,777 patients with epithelial ovarian cancer from SEER database, and demonstrated that married patients had a better survival compared to unmarried patients with an overall 5-year survival 45.0% for married patients and 33.1% for unmarried patients^16^. Krongrad *et al*. reported that married patients with prostate cancer had a better survival single, divorced, separated or widowed ones^17^. In this study, we first reported that widowed patients were at high risk of cancer-specific mortality and marriage can be a protective prognostic factor in CSS.

Our finding showed that married group had higher survival rate in patients of TNM stage I, II and IV. Although this correlation between marriage and cancer was supported by previous studies^18–22^, the reasons were not fully understood. Unlike unmarried ones, married patients are more likely to receive standard treatments and social support. It has been reported that social support can increase 1-year survival of patients with metastatic breast cancer^23^, and mitigate the harmful physiologic effects of stress and restrain cancer progression through immunologic or neuroendocrine pathways^24–26^. In addition, marriage reflects better economic status, which can provide the better nursing and medical care^27^. Gomez *et al*. found that the differences in economic resources resulting in different survivals of cancer patients^28^. Also, healthy lifestyles have been shown among married population and married patients can have extra health care from spouses. Finally, married patients showed less distress, depression, and anxiety than unmarried counterparts^29–31^. Many neuroendocrine mediators and cytokines present in depression and stress were found to be related to cancer metastasis^32, 33^. In addition, marital status also affects the diagnosis and treatment of patients. It has been reported that married patients would have better prognosis because of diagnosis and treatment at the early stage^34–36^. As mentioned above, marriage is known as the most important social support. Lack of economic and psychological support provided by marriage may attribute to the poor survival outcomes in unmarried patients. Therefore, we suggest that more psychological care and social support are needed for unmarried patients with GBC, especially for who are diagnosed at late stage and without treatment.

We also found that old (age ≥ 60) and female patients had worse prognosis. It might because aging would impair immune response, increase oxidative stress, shorten telomeres, and cause accumulation of senescent cells^37, 38^. While elder females experienced the changes of estrogen and progesterone which are closely related to the progression of cancer^39, 40^.

This study has several potential limitations. First, the SEER database does not include therapeutic information such as radical resection, palliative therapy, and detailed information of chemotherapy, recurrence and metastasis, which may also impact the prognosis of GBC patients^4^. Second, information of education, economic, social status and quality of marriage is not provided by this database, which would also effect on the prognosis of patients^12^. Third, marital status is not followed up after diagnosis, which may not be the real marital status of patients.

In conclusion, we found that married GBC patients had a higher 5-year cancer-specific survival (CSS) than that of unmarried ones. Widowed patients were at a high risk of cancer-specific mortality and marriage can be a protective prognostic factor in CSS.

## Method

### Patients

Data was obtained from the SEER database. The current SEER database consists of 18 population-based cancer registries that represent approximately 26% of the population in the United States. The SEER data contain no identifiers and are publicly available for studies of cancer-based epidemiology and health policy.

The National Cancer Institute’s SEER*Stat software (Surveillance Research Program, National Cancer Institute SEER*Stat software, www.seer.cancer.gov/seerstat (Version 8.3.2) was used to identify patients who were pathologically diagnosed as GC between 2004 and 2013 with single primary GBC and a known marital status of age ≥ 18. Histological types were limited to adenomas adenocarcinomas, epithelial neoplasms, cystic, mucinous and serous neoplasms, and unspecified neoplasms and others (squamous cell carcinoma/complex epithelial neoplasms/complex mixed and stromal neoplasms/ductal and lobular neoplasms). Patients were excluded if they had multiple primary malignant neoplasm, with distant metastasis (M1), died within 30 days after surgery or unavailable information of CSS and survival months.

### Statistical analysis

Clinicopathological parameters were analyzed by chi-square (χ2) test. Survival curves were generated using Kaplan-Meier estimates, and the differences were analyzed by log-rank test. Cox regression models were built for analyzing the risk factors of survival outcomes. Statistical analyses were performed using the statistical software package SPSS (version 19.0, Inc, Chicago, IL, USA). Results were considered to be statistically significant when a two-sided p values of less than 0.05.
